# Proposed Diagnostic Criteria for Misophonia: A Multisensory Conditioned Aversive Reflex Disorder

**DOI:** 10.3389/fpsyg.2017.01975

**Published:** 2017-11-14

**Authors:** Thomas H. Dozier, Michelle Lopez, Christopher Pearson

**Affiliations:** ^1^Misophonia Institute, Livermore, CA, United States; ^2^Department of Psychology, Alliant International University, San Diego, CA, United States; ^3^Center for Stress and Anxiety Management, San Diego, CA, United States; ^4^John Cox Health and Physiotherapy, West Yorkshire, United Kingdom

**Keywords:** misophonia, diagnostic criteria, sound sensitivity, neurodevelopmental disorders, anxiety, anger, classical conditioning, symptomatology

## Introduction

Misophonia is an underinvestigated condition often typified as an extreme sensitivity to specific, low volume sounds and images that elicit an intense physiological and emotional response (Jastreboff and Jastreboff, 2002, 2014; Edelstein et al., 2013; Schröder et al., 2013; Wu et al., 2014; Cavanna and Seri, 2015; Dozier, 2015a, 2017).

While misophonia has been described as a distinct psychiatric disorder that should be delineated as such (Schröder et al., 2013), Taylor (2017) proposed that extensive research is needed before such a conclusion is warranted. Symptomatology of misophonia intersects to some extent with that of several other recognized psychiatric disorders including but not limited to: specific phobia, obsessive compulsive disorder (OCD), sensory processing disorder (SPD), and posttraumatic stress disorder (PTSD). However, misophonia symptoms are not sufficiently captured by any diagnosis in the Diagnostic and Statistical Manual of Mental Disorders-5 (DSM-5; American Psychiatric Association, 2013; Bernstein et al., 2013). For a description of diagnostic differentials, the reader is referred to (Schröder et al., 2013). We support Taylor's (2017) call for research to accurately characterize misophonia as a disorder or features of another syndrome, but we also propose there is a pressing need to include the findings of recent research in diagnostic criteria for misophonia, regardless of whether misophonia is a distinct psychiatric, neurological, medical, or behavioral disorder, or a component of another disorder.

Although the prevalence of misophonia has yet to be formally determined, research suggests that it is not a rare condition. In a study that included a nonclinical sample of 483 undergraduate students, nearly 20% experienced clinically significant misophonia symptoms (Wu et al., 2014). In another study Schröder et al. (2017) reported that in a recent 5-year period, the center had over 500 misophonia patient referrals, further highlighting the prevalence of misophonia and the need to establish an accurate description of this condition. Collectively, the 3 authors have treated over 600 patients in the past 4 years. Presently, there are several relevant Facebook groups, of which one, the Misophonia Support Group, has over 15,000 members.

## Misophonia diagnostic criteria issues

Establishment of standardized diagnostic criteria is needed to increase recognition, advance empirical science, and inform clinical practice (Schröder et al., 2013; Cavanna and Seri, 2015; Taylor, 2017), including assessment and treatment of misophonia. Schröder et al. (2013) proposed diagnostic criteria for misophonia as a distinct psychiatric disorder based on an extensive evaluation of 42 patients. Subsequent research identifies several areas of clarification of misophonia which should be incorporated in a revision to the Schröder et al. (2013) criteria.

1. A misophonic stimulus can be produced by any source (i.e., human, animal, electronic, equipment, etc.; Edelstein et al., 2013; Wu et al., 2014; Cavanna and Seri, 2015; Dozier, 2015b, 2017; Dozier and Morrison, 2017; Taylor, 2017) and not limited to stimuli from humans.
2. A misophonic stimulus can be virtually any sensory modality, with auditory and visual stimuli being the most common (Bernstein et al., 2013; Edelstein et al., 2013; Johnson et al., 2013; Schröder et al., 2013; Cavanna and Seri, 2015; Dozier, 2015a,b,c, 2017; Dozier and Morrison, 2017; Taylor, 2017) and not limited to auditory stimuli.
3. The eliciting stimulus is a conditioned stimulus and so excludes responses to innate sensitivities, such as those with sensory processing disorder or sensory over-responsiveness (Jastreboff and Jastreboff, 2002, 2014; Dozier, 2015b, 2017; Dozier and Morrison, 2017).
4. The strength of the response is controlled by the context and experience with the stimulus and not the physical characteristics of the stimulus. Therefore, a minimal intensity stimulus will elicit the response (Jastreboff and Jastreboff, 2002, 2014; Bernstein et al., 2013; Edelstein et al., 2013; Dozier, 2015b, 2017).
5. The response is elicited by a single or small number of minimal intensity stimulus instances, and is not the response from persistent, annoying stimuli (Jastreboff and Jastreboff, 2002, 2014; Dozier, 2015a,b,c, 2017).
6. The stimulus elicits an immediate physical response, which is often a skeletal muscle response, but can be any physical response (Dozier, 2015a,b,c, 2017; Dozier and Morrison, 2017).
7. A moderate duration of the stimulus (e.g., 15 s) elicits physiological arousal (Edelstein et al., 2013; Kumar et al., 2017).

## Proposed diagnostic criteria

Drawing from the existing literature and clinical experience, and derived from Misophonia: Diagnostic Criteria for a New Psychiatric Disorder by Schröder et al. (2013), we propose the following criteria for identification and characterization of misophonia.

- The presence or anticipation of a specific sensory experience such as a sound, sight, or other stimulus (e.g., eating sounds, breathing sounds, machine sounds, leg movement, vibration), provokes an impulsive, aversive physical and emotional response which typically begins with irritation or disgust that quickly becomes anger.
  - Although auditory and visual stimuli are the most common, the stimulus can be in any sensory modality.
  - The stimulus is a conditioned stimulus, excluding those responses in which the stimulus is unconditioned, eliciting an unconditioned physiological response (i.e., excluding sensory over-responsiveness or sensory processing disorder).
  - Where a single occurrence or a small number of stimulus instances cause the default response.
  - Where a minimal intensity instance of the stimulus will elicit the default response (e.g., low volume baby crying or quiet breathing). If a high intensity instance of the stimulus is necessary to elicit the response, then it does not support the diagnosis of misophonia, especially if the stimulus is uncomfortably loud or startling.
- The stimulus elicits an immediate physical reflex response (skeletal or internal muscle action, sexual response, warmth, pain, or other physical sensation). Note the physical response cannot always be identified, but the presence of an immediate physical response may be used to more clearly identify the condition as misophonia.
- A moderate duration of the stimulus (e.g., 15 s) elicits general physiological arousal (e.g., sweating, increased heart rate, muscle tension).
- Dysregulation of thoughts and emotions with rare but potentially aggressive outbursts. Aggressive outbursts may be frequent in children.
- The negative emotional experience is later recognized as excessive, unreasonable, or disproportionate to the circumstances or the provoking stressor.
- The individual tends to avoid the misophonic situation, or if he/she does not avoid it, endures the misophonic stimulus situation with discomfort or distress.
- The individual's emotional and physical experience, avoidance, and efforts to avoid cause significant distress or significant interference in the person's life. For example, it is difficult for the person to perform tasks at work, attend classes, participate in routine activities, or interact with specific individuals.

## Conclusion

A review of the existing literature combined with clinical experience provides strong support for the aforementioned criteria for precisely identifying misophonia in clinical and research contexts. To facilitate effective research, diagnosis, and treatment, we suggest it is important to recognize that misophonia is a multi-sensory phenomenon and that it includes both an immediate elicited physical response and emotional responses, in addition to broad accompanying physiological responses. Although there is a need for further research into the characteristics and etiology of misophonia, we propose that misophonia be conceptualized as a conditioned aversive reflex disorder.

There is a pressing need for research into its processes, which may include the neural mechanism for the development of the initial physical response of misophonia, specifically stimulus-response classical conditioning (Donahoe and Vegas, 2004). Similarly, there exists important uncertainties each requiring scientific investigation and these include the syndromal boundaries of misophonia as they are identified and perhaps the breadth of a disorder of which misophonia may be a feature (Taylor, 2017).

That said, our experience in clinical practice supports a conclusion that early intervention often provides opportunity for quicker, more effective remediation (Dozier, 2017). Advancing the recognition of misophonia through clear, precise criteria provides an immediate and necessary benefit for those seeking therapeutic intervention.

## Author contributions

Developed proposed diagnostic criteria: TD, ML, and CP. Wrote article: TD and ML. Edited article: CP.

### Conflict of interest statement

All authors provide treatment to individuals with misophonia. CP trains and supervises therapists working with misophonia cases. TD is the owner of several misophonia apps.
